# Correction to “Pathways to enhancing prenatal diagnosis of skeletal dysplasias”

**DOI:** 10.1002/pmf2.70494

**Published:** 2026-10-24

Wang, M. J., C. Jacobsen, J. Arnold, R. J. Wapner and D. Krakow. (2026). “Pathways to Enhancing Prenatal Diagnosis of Skeletal Dysplasias.” *Pregnancy*, 2: e70284. https://doi.org/10.1002/pmf2.70284.

In Table 3, the ratio/measurement in the 7th row is erroneously listed as **BPD:FL** but should be listed as **FL:BPD**.

We apologize for this error.

